# Primary Sclerosing Cholangitis-Autoimmune Hepatitis Overlap Syndrome: Significant Barriers in Liver Disease Diagnosis and Treatment Experienced by the Latino Community

**DOI:** 10.7759/cureus.36126

**Published:** 2023-03-14

**Authors:** Daniel A Guifarro, Diana De Oliveira-Gomes, Renato Beas, Marcel J Yibirin-Wakim, Eleazar E Montalvan-Sanchez

**Affiliations:** 1 Department of Medicine, National Autonomous University of Honduras, Tegucigalpa, HND; 2 Department of Medicine, University of Texas Southwestern Medical Center, Dallas, USA; 3 Department of Medicine, Indiana University School of Medicine, Indianapolis, USA; 4 Department of Medicine, Boston University School of Medicine, Boston, USA

**Keywords:** social determinants of health, healthcare disparities, liver transplant, overlap syndrome, autoimmune hepatitis, primary sclerosing cholangitis

## Abstract

Overlap syndrome (OS) is a term that comprises the presentation of multiple hepatic disease characteristics in the same patient, such as the presence of autoimmune hepatitis (AIH) features in addition to primary sclerosing cholangitis (PSC) or primary biliary cholangitis (PBC). Standard therapy for AIH is immunosuppression, while ursodeoxycholic acid is the preferred treatment for PBC. Additionally, liver transplantation (LT) may be considered in severe cases. Hispanics have been found to have a higher prevalence of chronic liver disease and develop more complications associated with portal hypertension at the time of listing for LT. Despite being the fastest-growing population in the USA, Hispanics have a higher probability of not receiving an LT due to issues with social determinants of health (SDOH). It has been reported that Hispanics are more likely to be removed from the transplant list.

We report a case of a 25-year-old female immigrant from a Latin American developing country who presented with symptoms consistent with worsening liver disease after years of inappropriate workup and late diagnosis due to barriers within the healthcare system. The patient had a history of unresolved jaundice and pruritus and presented with worsening of her previous symptoms and new onset abdominal distention, bilateral leg edema, and telangiectasias. Laboratory and imaging studies confirmed the diagnosis of AIH and primary sclerosing cholangitis (PSC-AIH syndrome). The patient was started on steroids, azathioprine, and ursodeoxycholic acid, showing improvement. Due to her migratory status, she could not receive an appropriate diagnosis and follow up with a single provider or healthcare institution, putting her at increased risk for life-threatening complications. Although medical management is the first step, the probability of future liver transplants exists. The patient is still undergoing liver transplant evaluation and completing a workup since she was found to have an elevated model for end-stage liver disease (MELD) score.

Even with the introduction of new scores and policies that aim to reduce disparities in LT, Hispanic patients are still at higher risk of being removed from the waitlist because of death or clinical deterioration compared to non-Hispanics. To this day, Hispanics have the highest percentage of waitlist deaths (20.8%) of all ethnicities and the lowest overall rate for undergoing LT. Understanding and addressing the causes that could contribute to and explain this phenomenon is essential. Increasing awareness of this problem is vital to promote more research on LT disparities.

## Introduction

Overlap syndrome (OS) refers to the variant presentations of cholestatic autoimmune hepatic conditions such as primary biliary cholangitis (PBC) and primary sclerosing cholangitis (PSC), in conjunction with features of autoimmune hepatitis (AIH) [1,2]. Regarding PSC, it is a progressive disorder that causes inflammation and scarring of the bile ducts, leading to fibrosis, strictures, and dilation of the biliary tree [3]. The usual presentation involves 30-40-year-old males with a history of inflammatory bowel disease. Patients typically present biochemical signs of cholestasis, and most of the time, they are asymptomatic. Elevated serum alkaline phosphatase is the characteristic biochemical finding in PSC, and serum aminotransferase levels usually are only slightly elevated [1]. Cholangiography studies such as endoscopic retrograde cholangiopancreatography and magnetic resonance cholangiopancreatography identify the features commonly found in PSC. Typical histological findings for PSC comprise periductal fibrosis, inflammation, and proliferation of the portal ducts with lymphocyte infiltration.

Positive auto-antibody titers are expected, including antinuclear antibody (ANA), smooth muscle antibody (SMA), antineutrophil cytoplasmatic antibody, and perinuclear pattern (pANCA) [1-4]. Patients with OS usually present with symptoms such as fatigue, arthralgias, and myalgias, with serologic and pathologic workups demonstrating features of AIH with coexisting biliary strictures compatible with PSC or PBC [1-4]. It remains unclear if OS are variants of the main autoimmune hepatopathies or completely different entities. A possible common genetic background is a proposed explanation for [1]. Moreover, the transition from one autoimmune hepatopathy to another has also been described [2].

There are no specific criteria for OS diagnosis. The International Autoimmune Hepatitis Group suggests that patients should be categorized based on the disease-predominating feature [1]. It is essential to mention that PSC classically presents with an abnormal cholangiogram; however, there is a less common variant called small duct-PSC which presents with clinical and biochemical features of PSC and a normal cholangiography, requiring a liver biopsy for diagnosis [5]. Although OS is not standard, the OS between PSC-AIH has been widely described in children and adolescents, with a higher prevalence in men [3,4,6]. 

Treatment for a PSC-AIH OS must address both conditions. Standard therapy is immunosuppression with corticosteroids and azathioprine, while ursodeoxycholic acid is sometimes combined [1]. If medical treatment for the primary needs is impractical or complications like cirrhosis develop, leading to end-stage disease, liver transplantation (LT) must be considered as the treatment of choice [1,2,7].

Patients that present with AIH-PSC overlap obtain remission less frequently and often die from hepatic failure while waiting for the LT [1]. Furthermore, the clinical features and progression of the disease and their response to treatment and outcomes are vastly different within various ethnicities. At the time of disease presentation, it is more common for Hispanics to show characteristics of advanced liver disease, including portal hypertension and its complications. Additionally, the response to ursodeoxycholic acid treatment is less effective in Hispanics, especially with a younger disease presentation [7]. 

Due to the low prevalence of OS, long-term outcomes need to be better characterized and further studied [4]. Although autoimmune hepatopathies have shown comparable graft and patient survival after transplant, AIH seems to have worse transplantation-free survival [3].

The prevalence of liver disease is not the same between different races. Hispanics have been shown to have a higher prevalence of chronic liver diseases, representing the seventh most common cause of death in this group. According to the CDC, whites have a 30% lower mortality rate than Hispanics due to end-stage hepatic diseases. Moreover, in other ethnic groups, such as non-Hispanic whites and African Americans, liver diseases are not even included in the top causes of death. Cholankeril et al. describe data on patients with PBC on the liver transplant waitlist, where it was found that African American and Hispanic patients were younger compared to other ethnicities and presented a higher model for end-stage liver disease (MELD) score in the transplant list. When listing for the LT, they were also found to give more frequent complications such as ascites, hepatic encephalopathy, and spontaneous bacterial peritonitis [7].

Significant racial disparities in LT access have been widely described, including lower LT referral, waitlist removal, and lower post-transplant survival [8,9]. Even though with the introduction of the MELD score in 2002, racial disparities in blacks for liver allocation have decreased, mortality is still approximately 26% higher among blacks [8]. Furthermore, it has been shown that Hispanics are more likely to have Medicaid insurance or no insurance, which has been related to poor waitlist outcomes [7,9]. According to national reports, people with private insurance are more likely to have LT. Disparities are not over only when placed on the transplant list, even then, Hispanics are 10% less likely to have LT and 3.4% more likely to die while they are on the waitlist compared to whites [8].

Given all the described disparities, we report a case of a Latino patient with a diagnosis of OS who experienced significant delays in healthcare due to multiple social determinants of health (SDOH). We have also conducted a review of the literature on LT in this community.

## Case presentation

A female patient in her 20s, originally from a Northern Central American Country with medical history only significant for a previous cholecystectomy, presented with mild jaundice and pruritus for several years since her last pregnancy. She had previous unknown studies in a different US state for a probable liver condition without significant findings. The patient traveled to her home country as she did not understand her diagnosis and sought further explanations. An MRI at that time in her home country showed signs of chronic liver disease, and she was scheduled for a liver biopsy, but due to immigration issues, she had to return to the US. The patient then attended an initial clinic appointment to establish care with a new primary care provider. She reported worsening jaundice, pruritus, new onset abdominal distention, bilateral leg edema, and telangiectasias.

**Figure 1:**
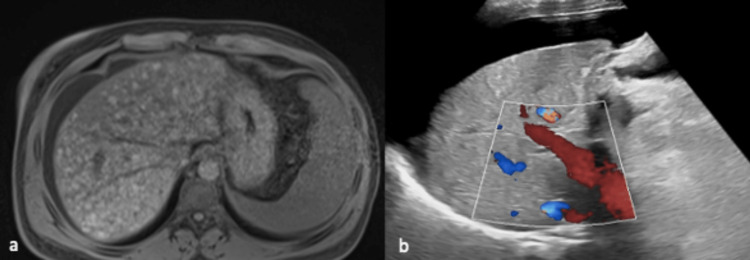
Abdominal MRI and ultrasound a. MRI of abdomen and pelvis T1 with contrast showing cirrhosis with multiple regenerative nodules. b. Abdominal US with Doppler showing patent hepatic vasculature.

The physical exam was positive for right upper quadrant (RUQ) tenderness and positive fluid wave. Laboratory studies were compatible with AIH/PSC overlap: aspartate aminotransferase (AST): 747, alanine aminotransferase (ALT): 546, alkaline phosphatase (ALP): 727, total bilirubin: 8.6 (direct 7.3), Gamma-glutamyl transpeptidase (GGT): 305, hepatitis viral panel: neg, IgG: 4700, antinuclear antibodies 1: 1280, antimitochondrial antibodies cytoplasmic pattern 1:80, mitochondrial antibodies: positive, alpha-1-antitrypsin QN 140 mg/dl, ceruloplasmin 36.1 mg/dl, vitamin D and vitamin A were low. An MRI showed cirrhosis with multiple regenerative nodules and ascites, and RUQ US showing patent hepatic vasculature (Figure 1). An upper endoscopy showed small esophageal varices and portal hypertensive gastropathy. 

The patient received treatment with ursodeoxycholic acid 600 mg Q12H (15 mg/kg/day divided into two daily doses), prednisone 40 mg daily, azathioprine 50 mg daily, aldactone 100 mg daily, and furosemide 40 mg daily with improvement. Liposoluble vitamins A, D, E, and K were also supplemented. Besides, a referral for LT evaluation was given due to the elevated MELD score on presentation to the clinic. After starting steroid treatment, the patient showed formal liver function tests, and she is currently on sirolimus as a steroid-sparing agent. The patient is still undergoing liver transplant evaluation and completing a preliminary workup.

## Discussion

**Figure 2:**
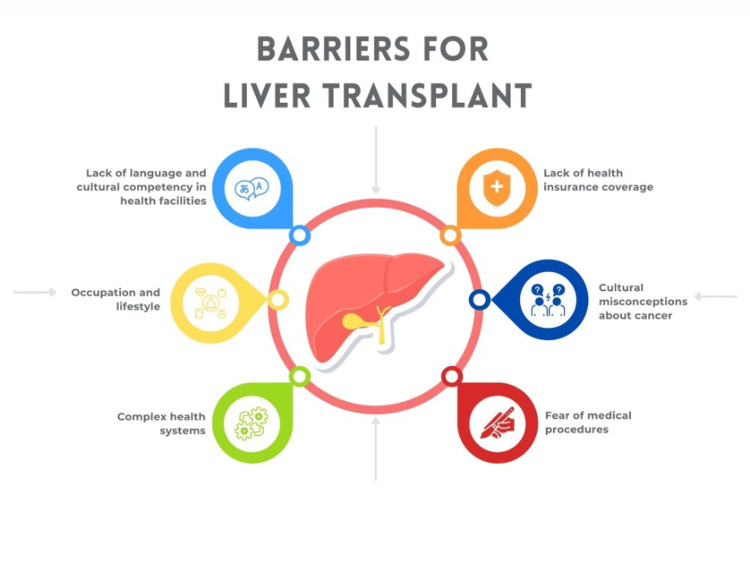
Barriers for liver transplant in the Latino community Image created by Eleazar Montalvan-Sanchez.

OS presents a disease with simultaneous features of two different autoimmune liver pathologies. In this case, our patient presented with PSC-AIH syndrome and faced many challenges jeopardizing her healthcare. Her history was deeply influenced by SDOH including lack of an insurance policy, language barriers as she can only speak Spanish, and receiving no continuity of care for her disease upon diagnosis (Figure 2). These SDOHs led the way to a delay in diagnosis and treatment, commonly faced by Hispanic patients, as shown in previous reports [3,7,10,11]. These factors convey more importance in the public health aspect of liver disease where Latinos have been shown to have higher disease prevalence and complications. 

The increased prevalence and severity of liver diseases ranging from nonalcoholic fatty liver disease to AIH, PSC, and PBC in the Latin American population are probably related to genetic predispositions and characteristics of this population, lack of access to health care, or the high prevalence of metabolic syndrome and its related diseases (insulin resistance, obesity, and diabetes) [12]. Hispanics have been shown to have higher mean triglyceride levels than other ethnicities, and the increased intrahepatic triglyceride levels are correlated with increased mitochondrial oxidative metabolism [12]. Socioeconomic factors also come into play in the Latino population as it has been reported that lower-income individuals have diets primarily comprised of fast foods and sugar-rich snacks and drinks. Data in the past has shown that 25% of Latin American adults live at or below the poverty line, being much higher than the 11% that has been reported for the non-Hispanic white population, and this is related to a more significant impact of SDOH like the language barriers and lack of access to care [12,13].

Despite being the fastest-growing population in the USA, Hispanics are more likely to be removed from the transplant waitlist due to death and less likely to be transplanted due to their SDOH. In addition, it has been reported that Hispanics have better post-liver transplant outcomes than non-Hispanics [8]. This is specifically important in the Latino population where it has been shown that liver diseases affect them disproportionately [12]. 

The MELD score has been implemented since 2002 for listing and recipient selection to decrease disparities in LT, ensuring that priority is given to the patients with the highest mortality risk. Due to the persistence of inequities, in 2013, a policy called “Share 35” (for patients with a MELD score ≥35) was implemented, aiming to reduce the number of deaths on the waitlist by increasing the regional sharing of livers. A study reviewing the outcomes of LT after “Share 35” found that Hispanic patients are still at higher risk of being removed from the waitlist because of death or clinical deterioration (SHR 1.23, 95% CI 1.13-1.34; P < 0.001), compared to non-Hispanics [14]. 

Moreover, Cholankeril et al. found that from 2000 to 2014, the number of Hispanic waitlist registrants with PBC was stable, while white additions decreased significantly, resulting in an increase in the proportion of Hispanic patients with PBC on the LT waitlist (from 10.7% in 2000 to 19.3% in 2014). Additionally, they reported that the highest percentage of deaths among all the ethnicities on the transplant waitlist was noted in the Hispanic patients (20.8%) who also had the lowest rate of transplantation (44.5%, compared to 62.2% in African Americans) and a higher mean MELD score in transplant recipients compared to other ethnicities (25.8; SD ± 10.3) [6]. The median waitlist time to transplant for Hispanics was 180 days, with a waitlist time to death being 308 days (higher and lower than the overall population, respectively). It was also described that in comparison to Whites, African Americans, and Hispanics more commonly had Medicaid insurance. Hispanics also were found to have the lowest literacy of the studied groups [14].

The World Health Organization establishes that racial disparities are unfair, unjust, unnecessary, and avoidable [15]. Understanding and addressing the causes that could contribute to and explain this phenomenon is essential. Increasing awareness of this problem is vital to promote more research on LT disparities.

There are significant racial disparities in LT. Hispanics are more likely not to receive a liver transplant and be removed from the list even though they have been found to have a greater prevalence of liver diseases and their complications with multiple environmental and genetic factors impact the outcome in this population. To this day, complications of the disease continue to cause death in patients while they are waiting on the transplant list. Despite implementing new policies, important socioeconomic factors affect LT disproportionately, affecting minorities such as Hispanics. These disparities cause delay in diagnosis and treatment of liver disease. 

It is necessary to propose and find solutions for this social health problem. The most crucial step to accomplish a fundamental change would be to recognize the race and ethnic disparities in healthcare and commit to reducing inequity. Proposed solutions to improve disparities in LT may include developing culturally competent interventions to improve health literacy in Hispanic patients, including public awareness campaigns, educational materials in Spanish, and support programs for patients and their families upon diagnosis of liver disease. Increased access to medical insurance and healthcare also plays a vital role in resolving this problem. Moreover, from a health professional perspective, it is essential to create programs to reduce implicit bias, deliver prompt referrals of Hispanic patients for a liver transplant, and implement strategies in healthcare agencies to reduce racial/ethnic disparities.

## Conclusions

Hispanics have been found to have a greater prevalence of liver diseases with multiple environmental and genetic factors that impact the outcome in this population. Additionally, critical socioeconomic factors affect LT which tends to affect minorities such as Hispanics disproportionally. These disparities delay in diagnosis and treatment of liver disease. Hispanics also have a higher mortality rate due to end-stage hepatic conditions when compared to whites. To this day, Hispanics are less likely to receive LT due to disease complications and death that may occur while waiting on the transplant list. Despite implementing new policies, racial disparities in LT remain an essential public health issue. It is necessary to understand the causes of these disparities and propose solutions to improve the access of the Hispanic community to LT and to surpass the problems with social determinants of health that the Latino community faces. These solutions should include interventions that improve knowledge about liver disease in the Hispanic community and support programs for patients and their families upon diagnosis of liver disease. Increasing access to care and providing more options for obtaining medical insurance is also vital in the approach to resolving these issues. It is also essential for the medical community to acknowledge this situation to reduce implicit bias and deliver prompt referrals of Hispanic patients for an LT.
